# The Effectiveness of Online-Only Blended Cardiopulmonary Resuscitation Training: Static-Group Comparison Study

**DOI:** 10.2196/42325

**Published:** 2023-04-05

**Authors:** Kah Meng Chong, Hsiang-Wen Yang, Hsien-Chin He, Wan-Ching Lien, Mei-Fen Yang, Chien-Yu Chi, Yen-Pin Chen, Chien-Hua Huang, Patrick Chow-In Ko

**Affiliations:** 1 Department of Emergency Medicine National Taiwan University Hospital Taipei City Taiwan; 2 Taiwan SaveANNE Education Association Taipei City Taiwan; 3 Department of Family Medicine National Taiwan University Hospital Taipei City Taiwan

**Keywords:** basic life support, cardiopulmonary resuscitation education, distance learning, self-directed learning, deliberate practice, blended learning, digital health intervention, virtual learning, virtual education, online course, digital training, online learning, classroom-based blended learning, educational outcomes, remote education, remote learning, static-group comparison study, online lectures, cardiopulmonary resuscitation, resuscitation

## Abstract

**Background:**

Basic life support (BLS) education is essential for improving bystander cardiopulmonary resuscitation (CPR) rates, but the imparting of such education faces obstacles during the outbreak of emerging infectious diseases, such as COVID-19. When face-to-face teaching is limited, distance learning—blended learning (BL) or an online-only model—is encouraged. However, evidence regarding the effect of online-only CPR training is scarce, and comparative studies on classroom-based BL (CBL) are lacking. While other strategies have recommended self-directed learning and deliberate practice to enhance CPR education, no previous studies have incorporated all of these instructional methods into a BLS course.

**Objective:**

This study aimed to demonstrate a novel BLS training model—remote practice BL (RBL)—and compare its educational outcomes with those of the conventional CBL model.

**Methods:**

A static-group comparison study was conducted. It included RBL and CBL courses that shared the same paradigm, comprising online lectures, a deliberate practice session with Little Anne quality CPR (QCPR) manikin feedback, and a final assessment session. In the main intervention, the RBL group was required to perform distant self-directed deliberate practice and complete the final assessment via an online video conference. Manikin-rated CPR scores were measured as the primary outcome; the number of retakes of the final examination was the secondary outcome.

**Results:**

A total of 52 and 104 participants from the RBL and CBL groups, respectively, were eligible for data analysis. A comparison of the 2 groups revealed that there were more women in the RBL group than the CBL group (36/52, 69.2% vs 51/104, 49%, respectively; *P*=.02). After adjustment, there were no significant differences in scores for QCPR release (96.9 vs 96.4, respectively; *P*=.61), QCPR depth (99.2 vs 99.5, respectively; *P*=.27), or QCPR rate (94.9 vs 95.5, respectively; *P*=.83). The RBL group spent more days practicing before the final assessment (12.4 vs 8.9 days, respectively; *P*<.001) and also had a higher number of retakes (1.4 vs 1.1 times, respectively; *P*<.001).

**Conclusions:**

We developed a remote practice BL–based method for online-only distant BLS CPR training. In terms of CPR performance, using remote self-directed deliberate practice was not inferior to the conventional classroom-based instructor-led method, although it tended to take more time to achieve the same effect.

**Trial Registration:**

Not applicable.

## Introduction

### Background

Basic life support (BLS) education plays a critical role in improving a community’s awareness of sudden cardiac arrest (SCA) and bystander cardiopulmonary resuscitation (CPR) rates. The outbreak of emerging infectious diseases, such as COVID-19, has become an obstacle to promoting BLS education in most emergency medical service (EMS) systems worldwide [1], and public willingness to perform CPR [2] and attend BLS classes has been affected by social distancing rules [3].

Several evidence-based strategies have been proposed for promoting CPR education. For example, the American Heart Association (AHA) recommended self-directed learning and deliberate practice during the pandemic [4], whereas the European Resuscitation Council specifically recommended distance learning [5]. In settings where face-to-face learning is restricted, distance learning may be useful for delivering CPR training [6-8].

In fact, distance learning (synonymous with online learning or e-learning) was proposed for CPR training as early as 2006 [9]. To date, AHA Heartsaver courses provide two different formats for distance learning: (1) an online-only course called Heartsaver Virtual [10] and (2) a blended learning (BL) course, in which a student completes part of the course online in a self-directed manner, followed by an instructor-led hands-on skills session [11]. In a systematic review, Elgohary et al [12] reported that the BL method was at least as effective as conventional CPR training methods, if not more so. Furthermore, Han et al [7] demonstrated that an online-only BL course could improve laypersons’ CPR skills to the same extent as a classroom-based conventional CPR training course. However, evidence regarding the practical effects of online-only CPR training courses is still scarce [13,14], especially evidence from comparative studies of online-only BL and classroom-based BL (CBL).

In addition, deliberate practice is a training approach in which learners are given the following: (1) a discrete goal to achieve, (2) immediate feedback on their performance, and (3) ample time for repetition to improve their performance [4]. A scoping review by Donoghue et al [15] suggested integrating deliberate practice instructional design models to enhance BLS courses and improve educational outcomes. Using deliberate practice models during conventional CPR training improves skill acquisition and retention in many critical tasks [4]. Hence, the effect and feasibility of deliberate practice in distance learning has not been studied and needs to be clarified.

### Objectives

To overcome the challenges in CPR education brought about by COVID-19, a novel online-only BL-based BLS course that implements distance learning and self-directed deliberate practice was developed to train the following: bystander CPR rate, automated external defibrillator (AED) use, and timely EMS activation for out-of-hospital cardiac arrest. The aim of this study is to describe the development of a novel online-only BLS training model—remote practice BL (RBL)—and compare its effect on learners’ performance with that of a conventional CBL course in an educational setting. We hypothesized that the RBL method would be noninferior to the CBL method in terms of the BLS sequence and CPR performance.

## Methods

### Study Design

A static-group comparison study was conducted to compare the effects of RBL-based BLS courses with CBL-based courses on learners’ performance in an educational setting.

### Study Setting

This study was conducted during the COVID-19 nationwide level 3 epidemic alert in Taiwan by SaveANNE Education, a qualified BLS training institute that hosts over 250 BL-based BLS training courses annually.

### Description of Conventional CBL

The CBL-based BLS courses at SaveANNE Education have 3 main parts. Part A is an online lecture session with 7 knowledge-related instructional videos that focus on first aid, EMS, BLS, CPR, and AED, followed by a mandatory online test with 20 multiple-choice questions (MCQs; Multimedia Appendix 1). Part B is an instructor-led hands-on deliberate practice session in which learners are given the following: (1) 3 discrete skill-related goals, including chest compressions, operating an AED, and adult BLS sequences; (2) immediate instructor and manikin feedback on their performance; and (3) ample time for repetition to improve their performance [4]. Part C is an on-site final assessment session, whose passing standard implies mastery of the learned skills. In the assessment session, learners are certified as meeting the following two criteria: (1) performing the BLS sequence correctly and (2) achieving at least 80% (80/100) in Little Anne QCPR scores for compression release, depth, and rate.

The Little Anne QCPR manikin (Laerdal Co) and its QCPR training app were used to show learners real-time and summative feedback on CPR compression performance, paired with AED Practi-TRAINER Essentials (WNL Products). The manikin to learner and instructor to learner ratios were 1:1 and 1:4, respectively. All qualified BLS instructors were paramedics with more than 3 years of practical EMS experience. All teaching materials, including instructional videos, MCQs, skill-training scenarios, and assessment criteria, were developed by the SaveANNE Education core team, comprising 2 experienced paramedics, 1 emergency physician, 1 EMS medical director, and 1 professional filmmaker.

### Description of RBL

**Figure 1:**
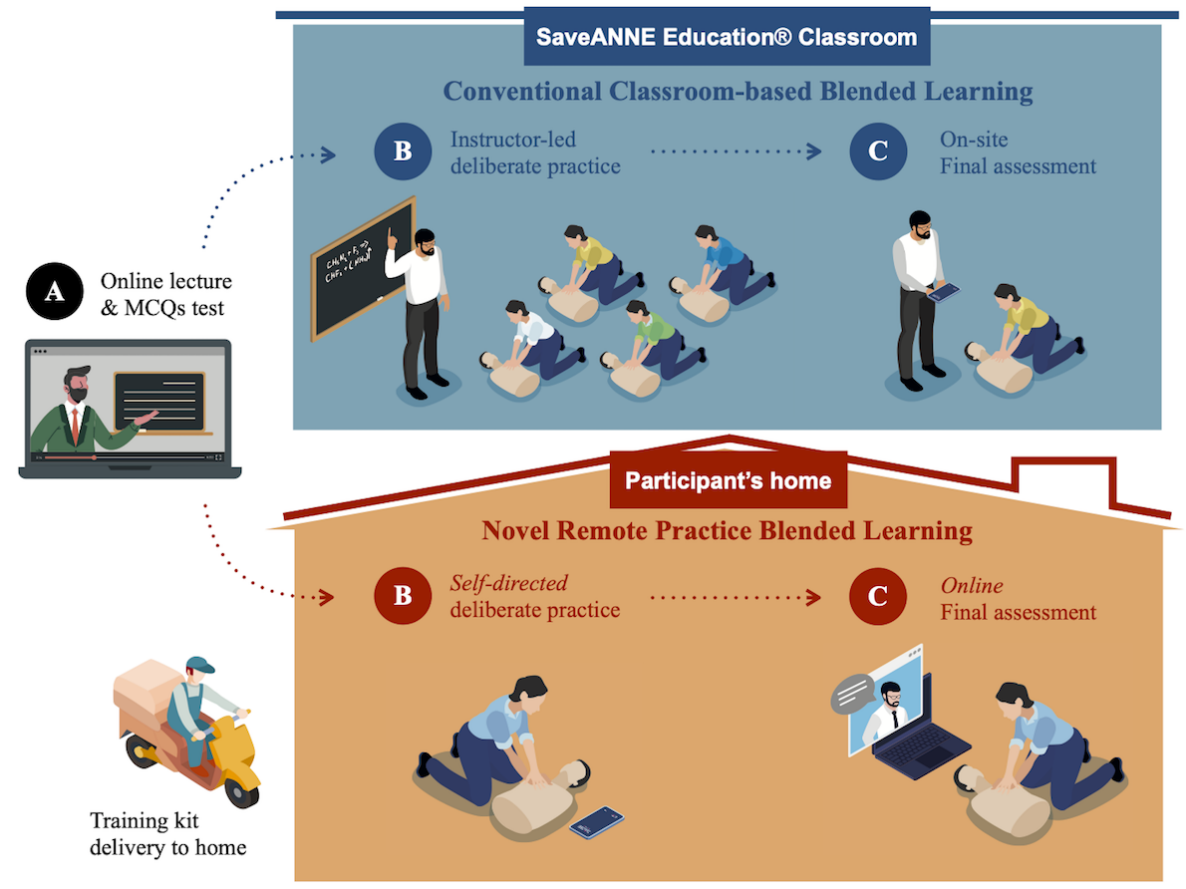
The transition from classroom-based blended learning to remote practice blended learning for a basic life support course. MCQ: multiple-choice question.

In May 2021, the Taiwan Central Epidemic Command Center raised the nationwide COVID-19 epidemic alert to level 3. As a result, most on-site educational facilities, including SaveANNE Education training classes, were suspended. Therefore, the CBL-based BLS course was modified into a new RBL-based BLS course, which also comprised 3 parts. Part A of the RBL-based BLS course was the same as the CBL-based course. Part B was modified to be a self-directed remote deliberate practice session that followed principles that were similar to the CBL-based course: (1) 3 identical discrete skill-related goals, including chest compressions, operating an AED, and adult BLS sequences; (2) immediate manikin-driven feedback on chest compression performance; and (3) ample time for repetition to improve performance [4]. Part C, the final assessment session, was conducted online and not in a classroom. The instructor evaluated the learners via video conference using the same scenario and passing criteria as in the CBL-based BLS course (Figure 1).

In the final assessment session of both the CBL and RBL-based BLS courses, the instructor had the opportunity to give feedback on learners’ BLS-related knowledge, skills, and attitudes, and each of the learners had the opportunity to repeat the final assessment session until they passed.

### Participants

During the study period, SaveANNE Education used the internet to invite learners from 38 and 68 RBL and CBL-based courses, respectively, to join as study participants. The exclusion criteria for the participants were as follows: (1) informed consent was not obtained and (2) course participation was insufficient.

### Sample Size

The sample size was estimated based on previous preliminary data, with an expected mean chest compression score of 95 (SD 4.5) among CBL-based BLS course learners. A sample size of 27 in each group had a power of 0.8 to detect a difference of 5 in means, assuming that the common SD was 6.5, using a 2-tailed, 2-group *t* test with a *P*<.05 2-sided significance level.

### Interventions

**Figure 2:**
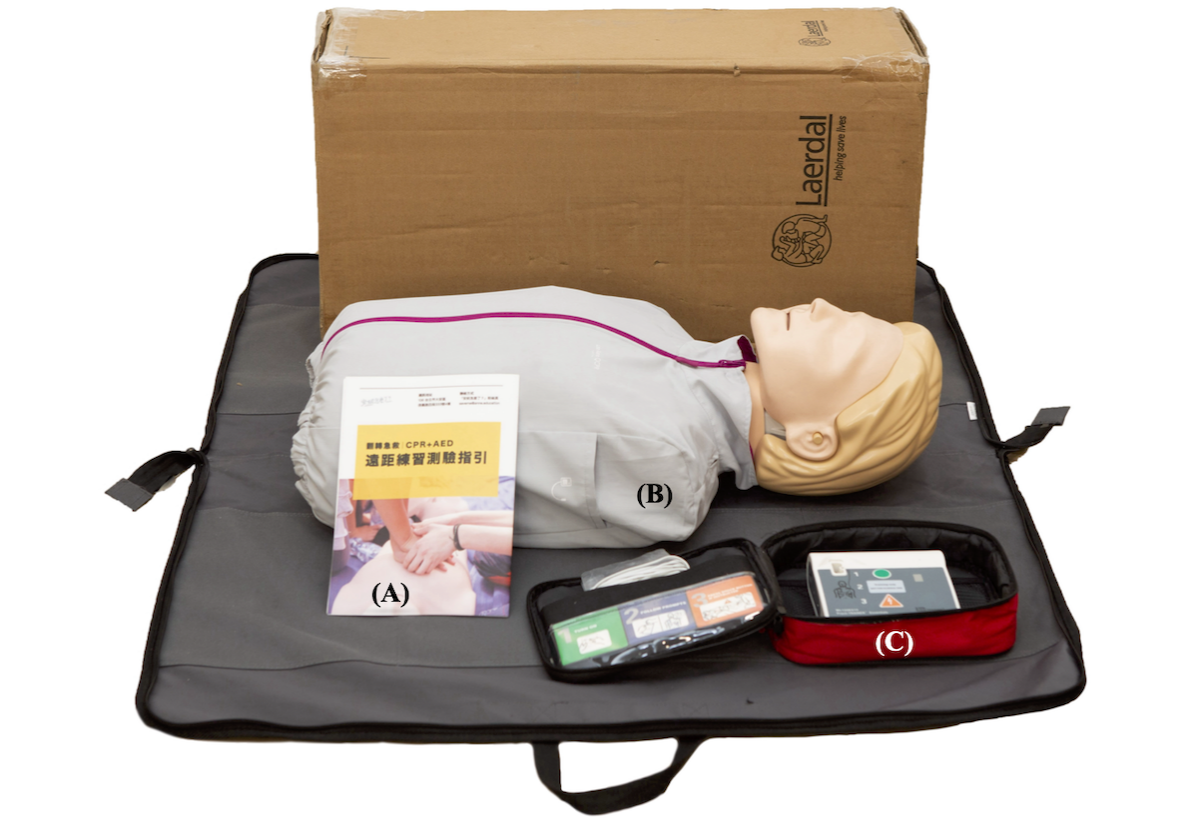
SaveANNE Education remote practice blended learning–based basic life support course training kit, which includes (A) a remote practice basic life support course manual, (B) a Little Anne quality cardiopulmonary resuscitation manikin, and (C) the set of Automated External Defibrillator Practi-TRAINER Essentials.

The participants in the experimental group were recruited during the nationwide COVID-19 level 3 epidemic alert, when only RBL-based BLS courses were available. As a first step, the participants were shown a web page where they watched the part-A instructional videos [16], following which they had to complete a mandatory MCQ test (Multimedia Appendix 1) off-site. When participants passed the online test, they were eligible to request a training kit (Figure 2) consisting of (1) remote practice BLS course material (Multimedia Appendix 2), (2) a Little Anne QCPR manikin, and (3) AED Practi-TRAINER Essentials course materials sent by SaveANNE Education, which were usually delivered within 3 days.

**Figure 3:**
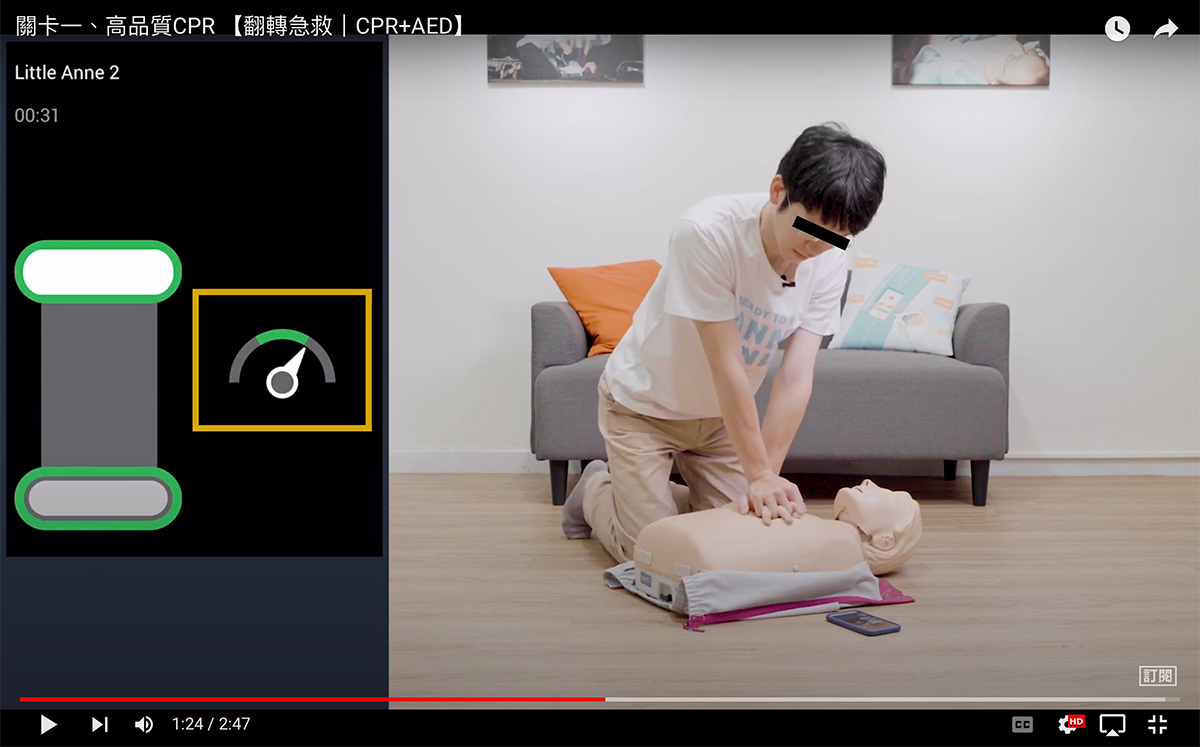
Screen capture of high-quality compression-only cardiopulmonary resuscitation instructional video.

After receiving the training kit, the participants could start remote deliberate practice. As shown in the practice manual (Multimedia Appendix 2), prior to self-practicing, participants were required to scan QR codes that linked to 3 specific, goal-directed, skill-related instructional videos (Figure 3) that described and demonstrated the essential skills of compression-only CPR, AED, and adult BLS sequences for laypersons in 12 minutes. Participants were encouraged to perform self-directed deliberate practice by repeatedly following these instructional videos until they were confident that they had mastered the requisite knowledge and skills. Then, they were free to schedule a final online assessment session with a SaveANNE Education instructor.

**Figure 4:**
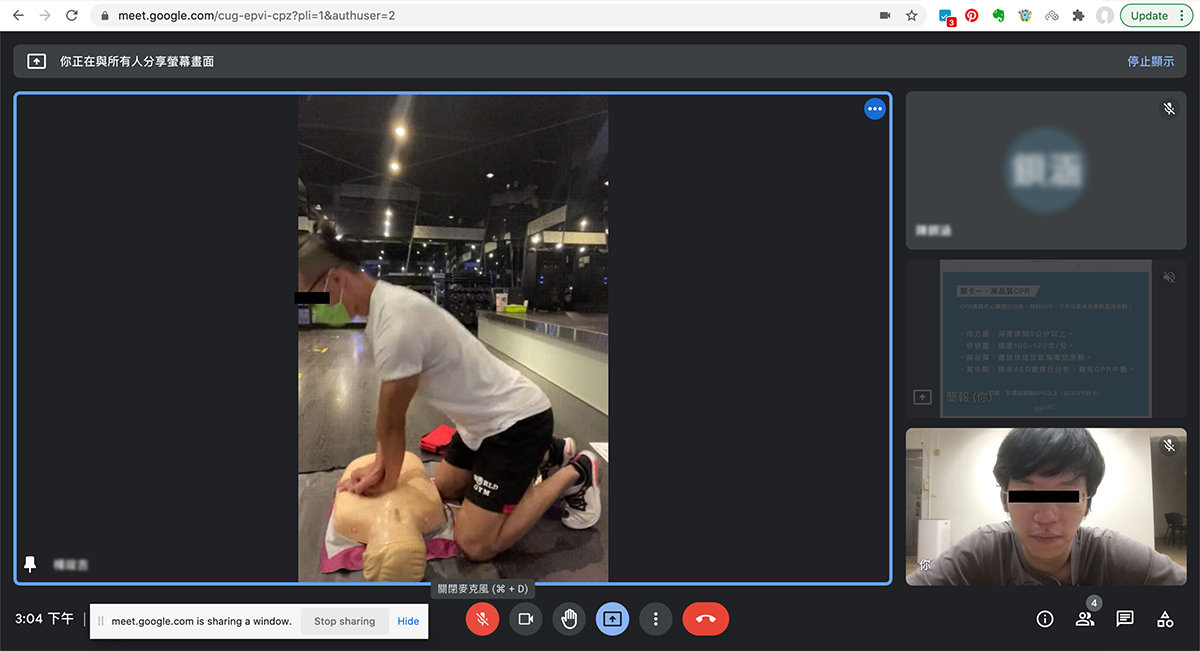
Screen capture of online final assessment session for the remote practice blended learning group.

The online final assessment session was conducted one-on-one via a Google Meet video conference between the instructor and participant (Figure 4). In this session, the participants were asked to handle a designated SCA scenario by performing the correct adult BLS sequence, high-quality CPR, and immediate AED operations on their manikin in front of the camera. Debriefing and feedback were provided by the instructor as needed to ensure that the criteria for passing were met by the participants, who also had the opportunity to repeatedly practice the SCA scenario until they passed. The length of the final online assessment session was determined by the debriefing duration and the number of retakes; it was expected to be less than an hour.

Finally, the study’s objectives and procedures were disclosed to the participants, who were invited to complete an online Google Form questionnaire consisting of 21 mandatory questions regarding the following: (1) age, gender, occupation, and motivation for attending the BLS course; (2) preexisting experience with CPR; (3) self-rated CPR performance before and after the self-directed deliberate practice session; (4) posttraining feedback regarding the difficulty of the BLS course, self-confidence and attitudes toward CPR and AED, and effectiveness of learning online, and (5) informed consent for participation in this study and provision of the studied information. For self-assessment questionnaires, we used a 5-point Likert scale ranging from 1 (totally disagree) to 5 (totally agree; Multimedia Appendix 3).

### Outcomes

The primary outcome was the Little Anne QCPR manikin–rated chest compression score. The secondary outcome was the number of retakes of the final assessment.

### Data Analysis

Statistical analyses were performed using SPSS (version 24; IBM Corp). Participants’ demographics and other characteristics were expressed as numbers and percentages. The QCPR manikin–rated chest compression score, 5-point Likert scale score, and time spent on courses were expressed as the mean (SD). The Shapiro-Wilk test was used to examine the normality of the data. Differences between the 2 groups were compared with the chi-square test for categorical variables and the nonparametric Mann-Whitney *U* test and a parametric independent-sample *t* test for continuous variables. Multiple linear regression analyses were conducted to evaluate the association between the BL modalities, deliberate practice measures, and study outcomes after adjusting for age, gender, occupation, and other variables that had been revealed as significant in the univariate analysis.

### Ethical Approval

The study design was approved by the National Taiwan University Hospital Institutional Review Board (202206110RINC).

## Results

### Inclusion of Study Participants and Their Characteristics

**Figure 5:**
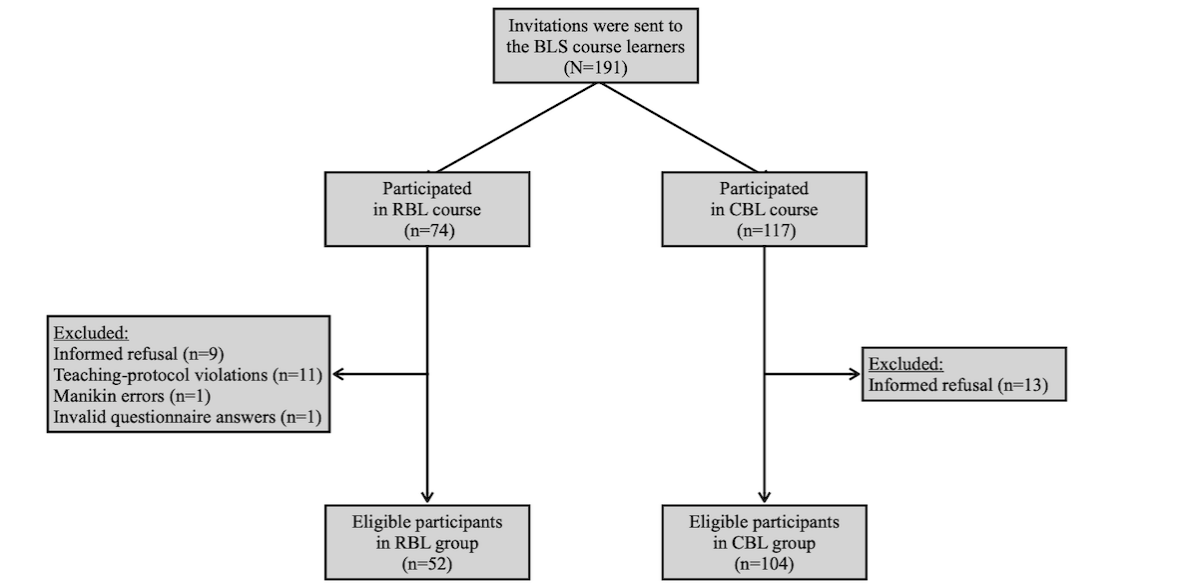
Flowchart of data collection. BLS: basic life support; CBL: classroom-based blended learning; RBL: remote practice blended learning.

During the study period, 191 online questionnaires, along with informed consent forms, were sent to the RBL- and CBL-based BLS course learners, comprising 74 and 117 participants in the RBL and CBL groups, respectively. After excluding 35 participants (35/191, 18.3%) owing to criteria such as informed refusal, teaching-protocol violations, manikin errors, and invalid answers to the questionnaire, 52 and 104 participants were eligible for data analysis in the RBL and CBL groups, respectively (Figure 5).

The participants’ characteristics are shown in Table 1. Ages, occupations, motivation for attending the BLS course, prior CPR training, and experience on humans were similar in the RBL and CBL groups, except that the RBL group had more women (36/52, 69.2% vs 51/104, 49%, respectively; *P*=.02).

In terms of learning performance, the RBL and CBL groups’ mean scores in the online lecture sessions were not significantly different (90.9 vs 91.8, *P*=.38). As for their skills-related performance, both groups passed the final assessment session by achieving high QCPR manikin–rated chest compression scores. The RBL and CBL groups had mean scores, respectively, of 96.9 and 96.4 (*P*=.58) for release of chest compression, 99.2 and 99.5 (*P*=.02) for depth of chest compression, and 94.9 and 95.5 (*P*=.66) for rate of chest compression.

However, the RBL group tended to spend more time practicing ahead of the final assessment session (12.4 vs 8.9 days, *P*<.001) and retook the final assessment session more times (1.4 vs 1.1 times, *P*<.001). The greatest number of RBL participants spent 30 to 60 minutes (21/52, 40.4%) in self-directed deliberate practice sessions, and nearly half of them (24/52, 46.2%) self-rated themselves as having average performance.

For posttraining self-evaluation, our results revealed most participants in the RBL and CBL groups agreed that their self-confidence for CPR and AEDs, and their willingness to perform CPR and AED on a stranger, improved after the training. The mean Likert-scale scores in the RBL and CBL groups for confidence for CPR and AEDs (4.75 vs 4.81, respectively, *P*=.62), willingness to perform bystander CPR (4.58 vs 4.57, respectively, *P*=.87), and willingness to use an AED on a stranger (4.83 vs 4.74, respectively, *P*=.68) were very high, and there was no significant difference between the 2 groups.

**Table 1:** Univariate analysis of characteristics of participants in the remote practice blended learning and classroom-based blended learning groups.

Characteristics		Remote practice blended learning group (n=52)	Classroom-based blended learning group (n=104)	*P* value	
**Age group (years), n (%)**					.64
	10-19	3 (5.8)	4 (3.8)		
	20-29	31 (59.6)	69 (66.3)		
	30-39	11 (21.2)	23 (22.1)		
	40-49	5 (9.6)	6 (5.8)		
	50-59	1 (1.9)	2 (1.9)		
	60-69	1 (1.9)	0 (0)		
**Gender, n (%)**					.02
	Male	16 (30.8)	53 (51)		
	Female	36 (69.2)	51 (49)		
**Occupation, n (%)**					>.99
	Health care provider	2 (3.8)	3 (2.9)		
	Non–health care provider	50 (96.2)	101 (97.1)		
**Motivation for attending basic life support course, n (%)**					.30
	Voluntary	11 (21.2)	30 (28.8)		
	Mandatory	41 (78.8)	74 (71.2)		
**Prior CPR^a^ training, n (%)**					.61
	Within last 6 months	3 (5.8)	2 (1.9)		
	Within 6-12 months	6 (11.5)	14 (13.5)		
	Within 12-24 months	5 (9.6)	15 (14.4)		
	More than 24 months	27 (51.9)	56 (53.8)		
	Never attended	11 (21.2)	17 (16.3)		
**Prior CPR experience on humans, n (%)**					.34
	Yes	2 (3.8)	9 (8.7)		
	No	50 (96.2)	95 (91.3)		
Online lecture session scores, mean (SD)		90.9 (6)	91.8 (6.3)	.38	
**Quality CPR chest compression scores, mean (SD)**					
	Chest compression release	96.9 (4.5)	96.4 (5.5)	.58	
	Chest compression depth	99.2 (1.4)	99.5 (1)	.02	
	Chest compression rate	94.9 (5)	95.5 (4.5)	.66	
Retakes of final assessment, mean (SD)		1.4 (0.6)	1.1 (0.3)	<.001	
Time spent before the final assessment (days), mean (SD)		12.4 (5.3)	8.9 (5.5)	<.001	
**Time spent on deliberate practice, n (%)**					
	Less than 30 minutes	9 (17.3)	N/A^b^	N/A	
	30-60 minutes	21 (40.4)	N/A	N/A	
	60-90 minutes	9 (17.3)	N/A	N/A	
	90-120 minutes	5 (9.6)	N/A	N/A	
	More than 120 minutes	8 (15.4)	N/A	N/A	
**Self-rating after deliberate practice, n (%)**					
	Far below average	0 (0)	N/A	N/A	
	Below average	1 (1.9)	N/A	N/A	
	Average (ie, meet passing criteria)	24 (46.2)	N/A	N/A	
	Above average	15 (28.8)	N/A	N/A	
	Far above average	12 (23.1)	N/A	N/A	
**Posttraining self-evaluation Likert-scale scores, mean (SD)**					
	Increased knowledge of CPR and AED^c^	4.94 (0.24)	4.86 (0.49)	.31	
	Increased conﬁdence for CPR and AED	4.75 (0.52)	4.81 (0.42)	.62	
	Increased willingness to perform CPR on a stranger	4.58 (0.60)	4.57 (0.67)	.87	
	Increased willingness to use AED on a stranger	4.83 (0.43)	4.74 (0.65)	.68	
	Refusal to perform basic life support on a stranger	1.73 (1.12)	1.81 (1.41)	.41	

^a^CPR: cardiopulmonary resuscitation.

^b^N/A: not applicable.

^c^AED: automated external defibrillator.

### Association Between BL Modalities and Study Outcomes in All Participants

Table 2 shows a multiple linear regression analysis of the association between BL modalities—RBL or CBL-based BLS courses—and outcomes after adjusting for potential confounding variables, such as participants’ age, gender, occupation, time spent before the final assessment session, and online lecture-session scores. In terms of primary outcomes, the RBL and CBL groups did not have significant differences in their QCPR manikin–rated chest compression release, depth, or rate scores: *P*=.61, *P*=.27, and *P*=.83, respectively. However, in terms of secondary outcomes, the number of retakes of the final assessment was significantly higher in the RBL than the CBL group (*P*<.001); a result that was similar to that of the univariate analysis (Table 1). In addition, a significant negative correlation was noted between the online lecture-session scores and the number of retakes of the final assessment (*P*=.04), which indicates that better lecture session scores were associated with fewer retakes of the final assessment.

**Table 2:** Multiple linear regression analysis of the association between blended learning modalities and their outcomes in all participants (n=156).

Outcome variables		β	*t* (*df*)	*P* value
**QCPR^a^ chest compression release score**				
	Constant		12.652 (6,149)	<.001
	Blended learning modalities^b^	.044	.516 (6,149)	.61
	Occupation^c^	.170	2.105 (6,149)	.04
	Gender^d^	–.080	–.96 (6,149)	.34
	Age	–.064	–.792 (6,149)	.43
	Time spent before the final assessment (days)	–.013	–.155 (6,149)	.88
	Online lecture session scores	.095	1.183 (6,149)	.24
**QCPR chest compression depth score**				
	Constant		65.720 (6,149)	<.001
	Blended learning modalities	–.095	–1.104 (6,149)	.27
	Occupation	–.044	–.544 (6,149)	.59
	Gender	.031	.377 (6,149)	.71
	Age	.077	.938 (6,149)	.35
	Time spent before the final assessment (days)	–.122	–1.445 (6,149)	.15
	Online lecture session scores	.015	.182 (6,149)	.86
**QCPR chest compression rate score**				
	Constant		14.222 (6,149)	<.001
	Blended learning modalities	.019	.222 (6,149)	.83
	Occupation	.063	.791 (6,149)	.43
	Gender	.071	.871 (6,149)	.39
	Age	–.109	–1.365 (6,149)	.17
	Time spent before the final assessment (days)	–.175	–2.121 (6,149)	.04
	Online lecture session scores	.153	1.931 (6,149)	.06
**Number of retakes of the final assessment**				
	Constant		3.437 (6,149)	.001
	Blended learning modalities	.347	4.375 (6,149)	<.001
	Occupation	.082	1.093 (6,149)	.28
	Gender	–.054	–.702 (6,149)	.48
	Age	.123	1.627 (6,149)	.11
	Time spent before the final assessment (days)	–.077	–.997 (6,149)	.32
	Online lecture session scores	–.152	–2.041 (6,149)	.04

^a^QCPR: quality cardiopulmonary resuscitation.

^b^For blended learning modalities, classroom-based blended learning was 0 and remote practice blended learning was 1 in all analyses.

^c^For occupation, health care provider was 0 and non–health care provider was 1 in all analyses.

^d^For gender, female was 0 and male was 1 in all analyses.

### Association Between Deliberate Practice Acquisition and the RBL Group’s Study Outcomes

Multiple linear regression analysis was also conducted to assess the association between self-directed deliberate practice acquisition and study outcomes in the RBL group (Table 3). After adjusting for potential confounding variables—age, gender, occupation, and time spent before the final assessment session—the analysis showed that the participants’ time spent on deliberate practice was significantly and positively correlated with their QCPR chest compression rate score (*P*=.047). Furthermore, RBL group participants who had a higher self-rating after deliberate practice were found to have significantly fewer retakes of the final assessment (*P*=.01). It is noteworthy that in this analysis, there was a significant negative correlation between time spent before the final assessment and the RBL participants’ QCPR chest compression rate score (*P*=.04); this correlation was also noted in all the RBL and CBL group participants in Table 2 (*P*=.04).

**Table 3:** Multiple linear regression analysis of the association between deliberate practice measures and outcomes in the remote practice blended learning group (n=52).

Outcome variables		β	*t* (*df*)	*P* value
**QCPR^a^ chest compression release score**				
	Constant		17.900 (6,45)	<.001
	Time spent on deliberate practice (hours)	.231	1.817 (6,45)	.08
	Self-rating after deliberate practice	–.125	–.909 (6,45)	.37
	Occupation^b^	.391	2.878 (6,45)	.01
	Gender^c^	–.053	–.384 (6,45)	.70
	Age	–.079	–.620 (6,45)	.54
	Time spent ahead of the final assessment (days)	–.112	–.865 (6,45)	.39
**QCPR chest compression depth score**				
	Constant		55.727 (6,45)	<.001
	Time spent on deliberate practice (hours)	.048	.328 (6,45)	.75
	Self-rating after deliberate practice	–.047	–.297 (6,45)	.77
	Occupation	–.050	–.318 (6,45)	.75
	Gender	.041	.257 (6,45)	.80
	Age	.111	.758 (6,45)	.45
	Time spent ahead of the final assessment (days)	–.224	–1.501 (6,45)	.14
**QCPR chest compression rate score**				
	Constant		15.512 (6,45)	<.001
	Time spent on deliberate practice (hours)	.267	2.042 (6,45)	.047
	Self-rating after deliberate practice	.174	1.240 (6,45)	.22
	Occupation	.068	.488 (6,45)	.63
	Gender	.176	1.248 (6,45)	.22
	Age	–.108	–.825 (6,45)	.41
	Time spent ahead of the final assessment (days)	–.280	–2.111 (6,45)	.04
**Number of retakes of the final assessment**				
	Constant		3.448 (6,45)	.001
	Time spent on deliberate practice (hours)	.028	.219 (6,45)	.83
	Self-rating after deliberate practice	–.432	–3.079 (6,45)	.01
	Occupation	–.037	–.269 (6,45)	.79
	Gender	.291	2.066 (6,45)	.045
	Age	.260	1.998 (6,45)	.052
	Time spent ahead of the final assessment (days)	–.155	–1.174 (6,45)	.25

^a^QCPR: quality cardiopulmonary resuscitation.

^b^For occupation, health care provider was 0 and non–health care provider was 1 in all analyses.

^c^For gender, female was 0 and male was 1 in all analyses.

## Discussion

### Principal Findings

This study made 3 major findings. First, it demonstrated a novel but feasible online-only BL-based BLS course design for implementing distance learning and self-directed deliberate practice. Second, it confirmed that this remote self-directed deliberate practice method was not inferior to conventional classroom-based instructor-led methods in terms of the BLS sequence and CPR performance. Third, it revealed that the RBL group needed more retakes to achieve the same performance as the CBL group at the end of the courses. These findings could be helpful in exploring innovative resuscitation education, which may shape better strategies or guideline modifications for enhancing bystander CPR achievements.

### Comparison to Prior Work

Our RBL-based BLS course design was derived from a literature review. Although previous studies have shown that online-only learning [6], conventional CBL [12], self-directed learning [17], and deliberate practice or mastery learning [15] can provide pragmatic and reasonably effective alternatives to conventional CPR training, none of them incorporated all these evidence-based instructional methods into a BLS course, as we have done and demonstrated in our pioneer study. To the best of our knowledge, this is the first comparative study to show that online-only BL is not inferior to CBL in terms of CPR educational outcomes.

Our study’s results have strengthened the evidence that the CPR performance of self-directed deliberate practice learners is similar to that of conventional instructor-led learners. A systematic review of 22 randomized trials comparing the effect of these 2 training methods in BLS courses also found that the most frequent conclusion of these trials was that self-directed courses had similar educational outcomes as instructor-led courses [17], which is consistent with our study results. Although it remains uncertain as to which of the 2 methods is superior, the AHA 2020 guidelines recommend considering the self-directed method as a reasonable alternative to instructor-led CPR training for lay rescuers [4].

As regards cost-effectiveness, it has been postulated that as a BL approach allows for some parts of the course material to be viewed and learned online, the overall in-person course can be shortened, which in turn reduces costs and the time that participants and faculty members spend in the classroom environment [12]. Another previous study suggested that self-directed learning is a more time-efficient tool for CPR training, as it reduces costs (eg, for instructors) [18].

Nevertheless, it has been pointed out that using these approaches is not necessarily cost-effective, and consideration should be given to the following: the type of BL, staff expertise, and the educational setting [19]. Reflecting on this point, it is noteworthy that in our study the cost of RBL courses was significantly higher than that of CBL courses, while their effects were similar. To achieve the same teaching quality and learning value, the manikin to learner and instructor to learner ratios in the RBL courses were optimized to 1:1. Other than that, our results revealed that the RBL learners spent more time than the CBL learners to complete the BLS courses, including the time spent before the final assessment (12.4 vs 8.9 days, respectively; *P*<.001) and the number of times they retook the final assessment (1.4 vs 1.1 times, respectively; *P*<.001). Based on practical considerations, we surmise that the RBL group may have spent additional time for three reasons: (1) waiting for the delivery of the training kit, (2) the process of video conferencing–based feedback from instructors, and (3) the need to retake the designated SCA scenarios to pass the examination. Hence, the RBL group needed to increase their time spent on courses to achieve the same performance as the CBL group at the end of the courses. To our knowledge, there is no prior research or evidence specifically emphasizing this point; therefore, our study fills a gap to a certain extent.

This study’s self-directed deliberate practice design in BL was a novel approach. In the RBL group, increased time spent on deliberate practice was significantly associated with a better QCPR chest compression rate score (Table 3), and participants with a higher self-rating after deliberate practice had significantly fewer retakes of the final assessment (Table 3). These findings demonstrate the influence and effectiveness of self-directed deliberate practice in BL, which could reinforce the idea that this novel approach is rational and useful.

Finally, it is of interest that we noted relatively poor CPR performance among RBL group participants, who spent more time before the final assessment session. We speculate that these learners may have been less aggressive in learning or faced problems with deliberate practice. Hence, more attention should be paid to these learners in future BL-based CPR education.

### Limitations

Our study had some limitations. First, it was a nonrandomized experimental study. Second, the participants and evaluators were not blinded to the interventions, given that it would have been difficult to do so in an educational study. Third, the sample size was small, although the number of participants permitted adequate power for the analyses. Fourth, we did not assess several aspects of BLS skills in specific scenarios, such as scene-safety checks, calls for help, and open airways. Fifth, as we used a questionnaire, we were only able to measure participants’ estimated time spent on deliberate practice, and not the actual time spent, in the RBL group. Sixth, considering the influence of the precondition that the participants had expertise in online learning, we were unable to determine the participants’ prior experience and familiarity with online learning, which could have influenced the study results. Seventh, we did not evaluate skill retention after a specific time period after the course. Long-term skill decay in the 2 cohorts over various periods of time (eg, 3, 6, or 9 months or longer) is unknown. Finally, as this pioneering study was conducted in a single institution, there could have been external variations in socioeconomic status, internet culture, software, and infrastructure settings. More studies are needed to fill knowledge gaps on the issue of CPR education.

### Conclusions

We developed a remote practice blended-learning method for online-only distant BLS course CPR training. Layperson CPR training using a remote self-directed deliberate practice method was not inferior to the conventional classroom-based instructor-led method in terms of BLS familiarity and CPR performance. Although this novel online-only BL method tended to take more time to achieve the same effect as conventional BL, we consider it to be a reasonable alternative CPR training method.

## Appendix

Multimedia Appendix 1 Multiple-choice questions after the online lecture.

Multimedia Appendix 2 CPR + AED blended learning: Remote practice BLS course material.

Multimedia Appendix 3 Questionnaire after the BLS course.

## Abbreviations

AED: automated external defibrillator
AHA: American Heart Association
BL: blended learning
BLS: basic life support
CBL: classroom-based blended learning
CPR: cardiopulmonary resuscitation
EMS: emergency medical service
MCQ: multiple-choice question
QCPR: quality cardiopulmonary resuscitation
RBL: remote practice blended learning
SCA: sudden cardiac arrest

## Data Availability

The data that support the findings of this study are available from the corresponding author, PCIK, upon reasonable request.
