# Supplementary material for: The Effectiveness of Online-Only Blended Cardiopulmonary Resuscitation Training: Static-Group Comparison Study
Source: J Med Internet Res. 2023 Apr 5;25:e42325. doi: 10.2196/42325 (PMC10131976; doi:10.2196/42325)
Supplement: Multimedia Appendix 1 [file jmir_v25i1e42325_app1.docx]

**Appendix 1:** **Multiple-choice questions after the online lecture**

1. What is the golden time to resuscitate a patient with sudden cardiac arrest?

A.   1—3 minutes

B.   4—6 minutes

C.   8—10 minutes

D.  12—15 minutes

2. When emergency medical services are activated, who should be the first responders in our country?

A.   Doctor

B.   Nurse

C.   Emergency medical technician (EMT)

D.  Policeman

3. When should CPR and AED be started?

A.   Patient is unconscious and breathing abnormally

B.   Patient is unconscious

C.   Patient is breathing abnormally

D.  Patient is having seizures

4. Which of the following are not indications of calling 119 ambulance services?

A.   Shortness of breath

B.   Cold sweat with acute chest tightness

C.   Fever without other symptoms

D.  Sudden weakness in the left upper and lower limbs

5. Which of the following statements about CPR are false?

A.   It pumps the blood from the heart to other major organs by compressing the chest

B.   It needs to be performed on a hard surface, such as a floor

C.   It supplies oxygen to the brain and heart

D.   It can replace more than 80% of the ejection fraction of a normal heart

6. Which of the following statements about AED are false?

A.   It can restore the rhythm of a standstill heart.

B.   It can restore the rhythm of a ventricular-fibrillating heart.

C.   It can automatically detect lethal arrhythmias

D.   It will not defibrillate a person with abnormal vital signs

7. The BLS for laypersons emphasizes that chest compression-only CPR (CCC) should be performed in which of the following scenarios?

A.   OHCA of children

B.   OHCA of drowning

C.   OHCA or acute myocardial infarction.

D.  OHCA due to choking asphyxia

8. Which of the following statements regarding abnormal breathing is false?

A.   Apnea should be considered as abnormal breathing.

B.   Chest rising should be considered as normal breathing.

C.   Signs of gasping should be considered as abnormal breathing.

D.  When in doubt, the 119 dispatcher’s instructions should be followed to evaluate breathing.

9. What is the best method for maintaining a chest compression depth of more than 5 cm?

A.   Using a ruler to measure the depth

B.   Pressing as gently as possible

C.   Pressing as hard as possible.

D.   Looking at the AED waveform

10.  What is the recommended CPR compression rate for adult patients?

A.   80—100 times/min

B.   100—120 times/min

C.   120—140 times/min

D.   140—160 times/min

11.  What is the recommended location for CPR compression in adult patients?

A.   Between the nipples

B.   Xiphoid process

C.   Below the left nipple

D.   Upper abdomen

12.  Where can we obtain public access to AED?

A.   Shopping centre

B.   School

C.   Sports centre

D.   All of the above

13.  Assuming that an old man suddenly collapses in front of you but you are not sure about his breathing condition, what is the most appropriate next step?

A.   Do nothing except wait for the ambulance.

B.   Call 119 immediately

C.   Do nothing except wait for him to wake up

D.   Ask for help from passersby

14.  Which of the following is the correct order in the adult OHCA chain of survival?

A.  EMS activation, early CPR, rapid defibrillation, advanced resuscitation, post-cardiac arrest care, recovery

B.  Early CPR, rapid defibrillation, advanced resuscitation, post-cardiac arrest care, recovery

C.   Early CPR, rapid defibrillation, EMS activation, advanced resuscitation, post-cardiac arrest care, recovery

D.  EMS activation, rapid defibrillation, early CPR, advanced resuscitation, post-cardiac arrest care, recovery

15.  Which of the following are the correct steps for using an AED?

A.  Attach the pads, turn on the AED, and plug the pad’s connector cable

B.  Plug the pad’s connector cable, turn on the AED, and attach pads

C.   Turn on the AED, attach the pads, and plug the pad’s connector cable

D.  Plug the pads connector cable, attach the pads, and turn the AED on.

16.  Which of the following is not an acceptable reason to pause CPR?

A.  An emergency medical technician (EMT) has arrived to take over CPR

B.  The patient has regained consciousness and is breathing normally

C.   The chest compressor is switching roles with another rescuer

D.  The CPR started 3 minutes back

17.  Which of the following statements about AED are false?

A.  Avoid touching the patient when the AED is defibrillating

B.  Avoid switching off the AED during CPR

C.   The placement of AED pads should be parallel to the heart axis

D.  The AED will re-analyze the rhythm every 5 minutes

18.  If a cardiac arrest patient does not receive CPR, by how much is the chance of survival reduced each minute?

A.  3%

B.  5%

C.   10%

D.  20%

19.  What is helpful when your friend suddenly collapses in front of you?

A.  119 Dispatcher’s pre-arrival instructions

B.  AED voice instructions

  BLS knowledge and skills

D.  All of the above

20.  What is the correct adult BLS sequence for a layperson?

A.  Check responsiveness (call the patient), activate EMS (call 119), compression, and defibrillation

B.  Check responsiveness (call the patient), activate EMS (call 119), compression, and airway

C.   Check responsiveness (call the patient), activate EMS (call 119), compression, airway, breathing, and defibrillation

D.  Check responsiveness (call the patient), activate EMS (call 119), airway, breathing, and compression
