# Supplementary material for: The Effectiveness of Online-Only Blended Cardiopulmonary Resuscitation Training: Static-Group Comparison Study
Source: J Med Internet Res. 2023 Apr 5;25:e42325. doi: 10.2196/42325 (PMC10131976; doi:10.2196/42325)
Supplement: Multimedia Appendix 2 [file jmir_v25i1e42325_app2.docx]

*
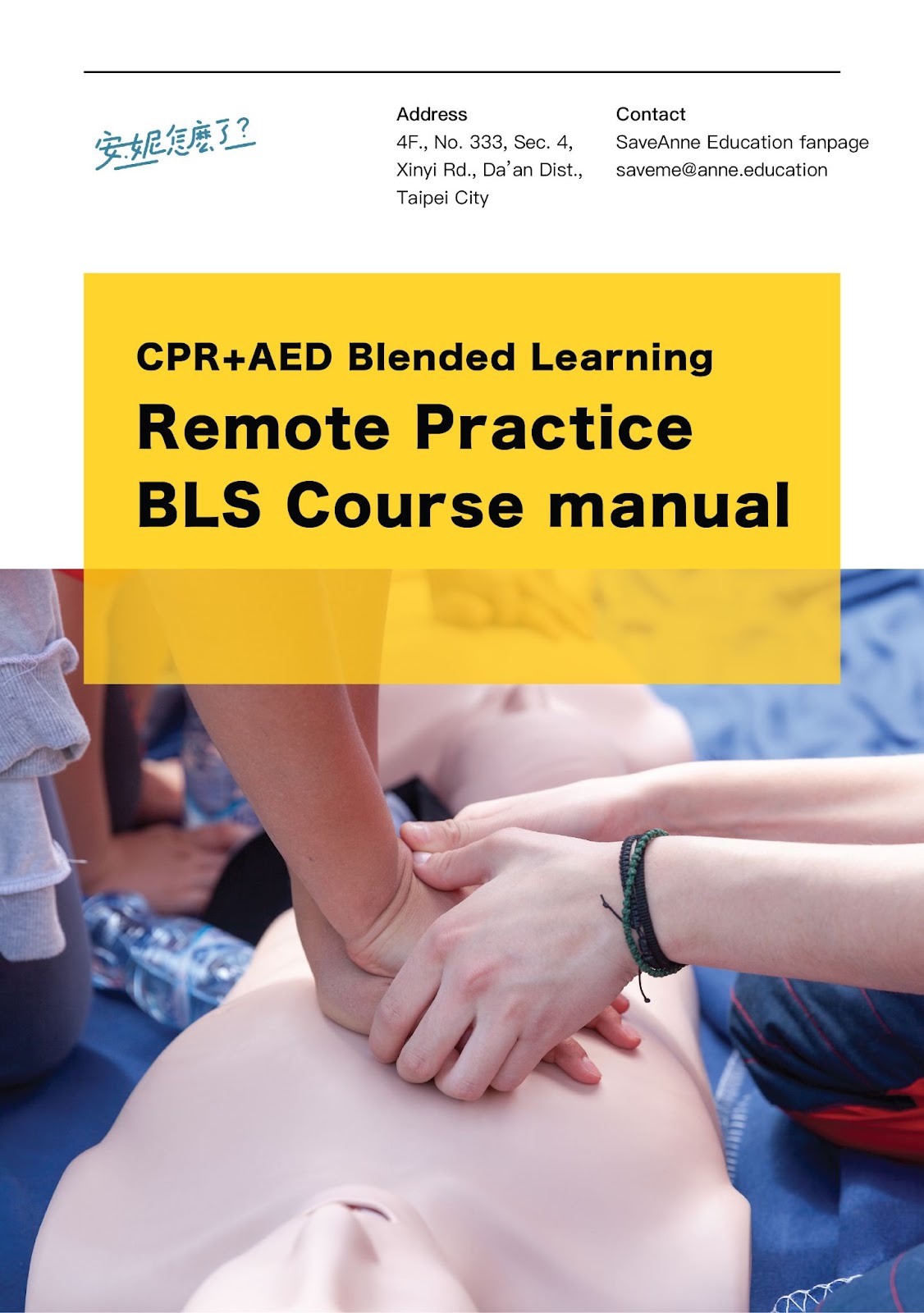
*

Thank you for participating in the “Remote Practice Blended Learning BLS course,” hosted by SaveANNE Education®. Ever since the COVID-19 pandemic, our conventional classroom-based blended-learning BLS course has been modified, using a fully online approach. To complete this BLS course, you must conduct self-directed deliberate practice in advance, using this manual, and schedule a final assessment session with the course instructor using Google Meet.


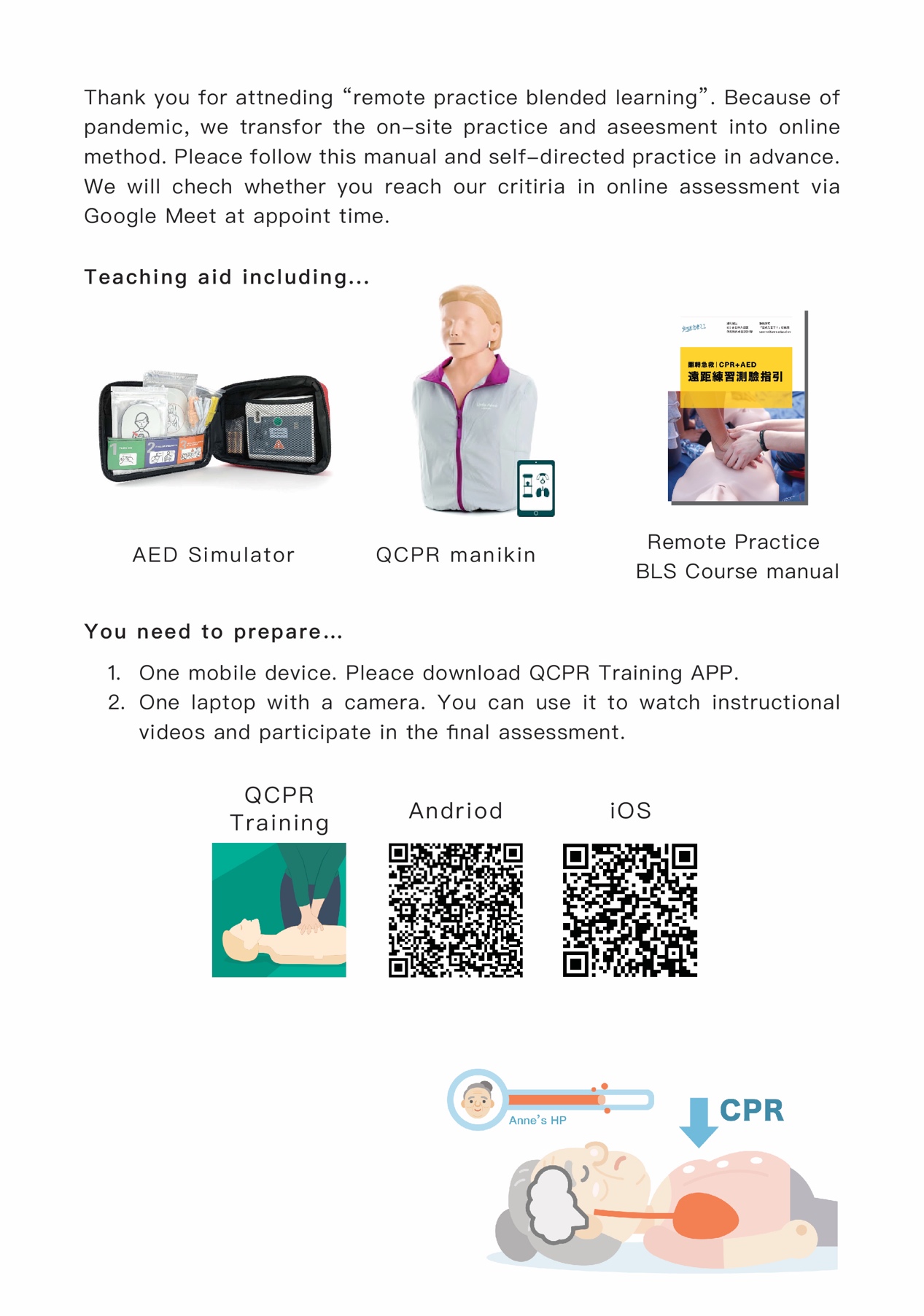


You will also need to prepare…

1. A mobile device with a downloaded QCPR training app (scan QR code).
2. A laptop with a camera for watching instructional videos as well as for video conferencing.


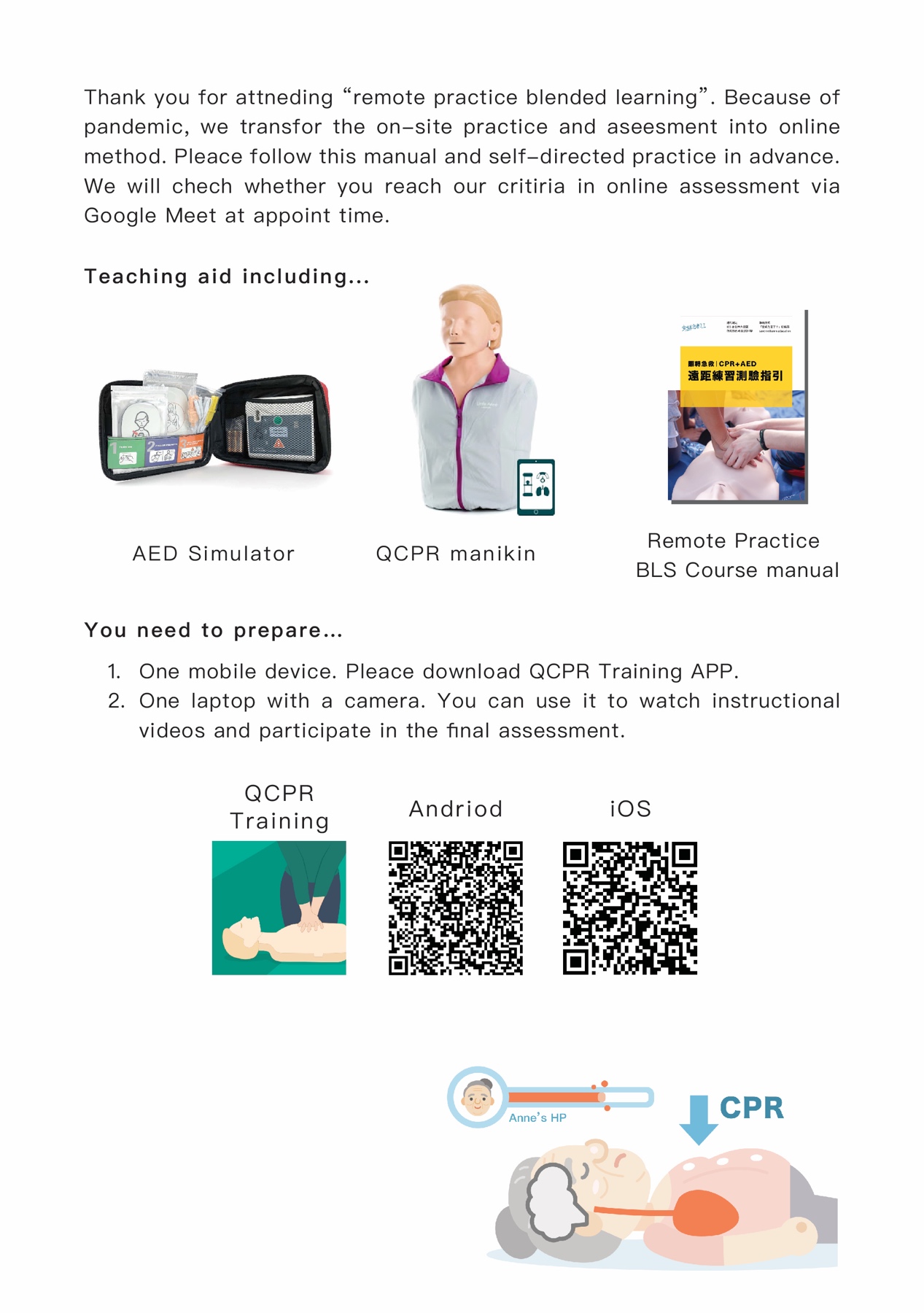


**Preparation**

The Laerdal Little Anne QCPR system will be used in our BLS training sessions. It comes with real-time feedback technology to help you improve the CPR quality and training efficiency. Please follow the setup steps listed below.

1. Activate the Little Anne manikin by doing a couple of chest compressions, until you hear a “beep” sound. The manikin automatically turns off if it is suspended for some time.
2. Open the Bluetooth and QCPR training apps on the mobile device.
3. Click the
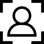
 profile icon in the upper-right corner for pairing it with the detected manikin.
4. Return to the home screen, set the compression duration to 1 min, and click “START” in the compressions-only panel (green).
5. Start chest compressions when ready. At the end of practice, the app will display CPR quality, including compression depth, release, rate, and total score.


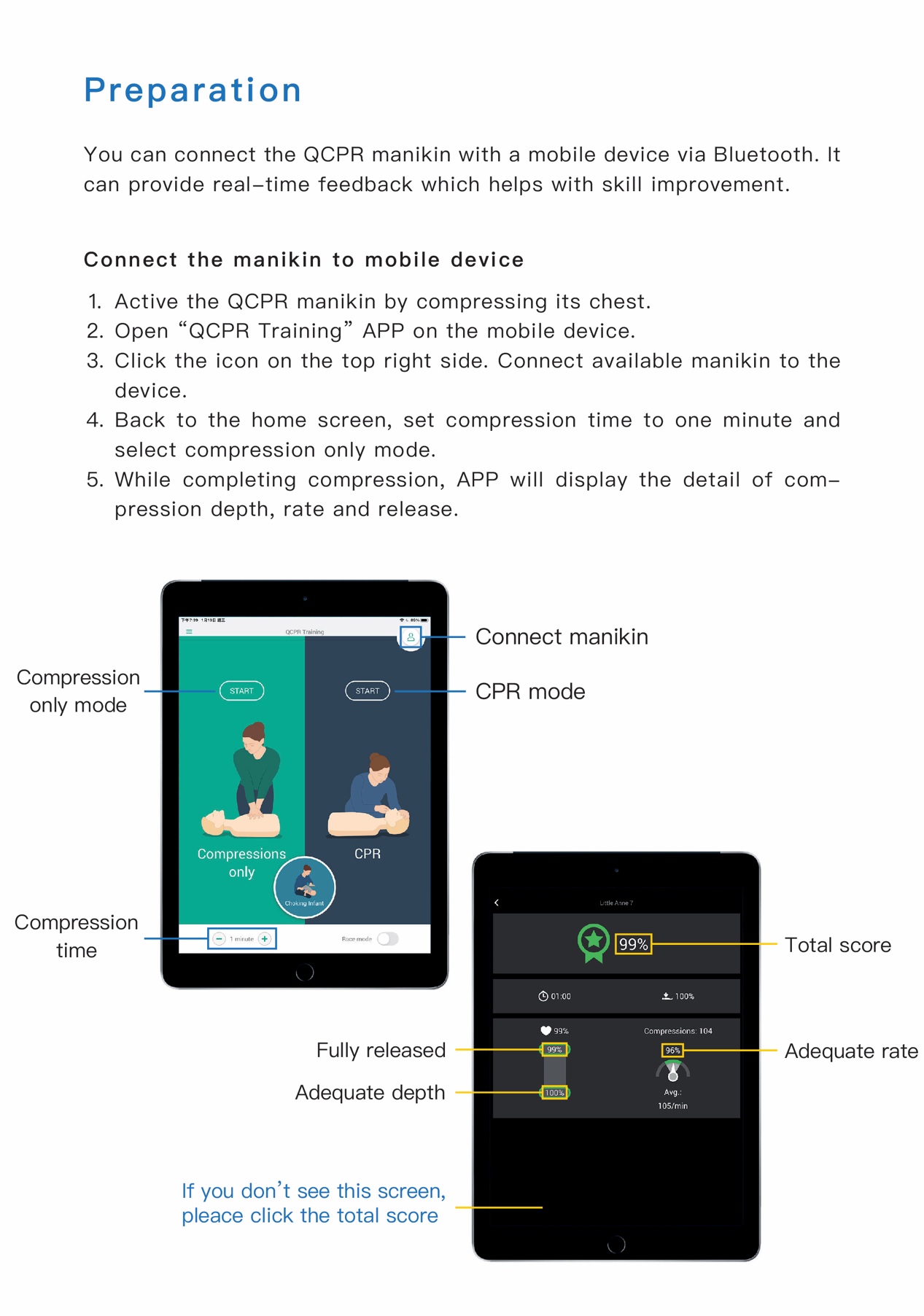


**Self-directed Deliberate Practice**

This session had three learning objectives, each of which, had an instructional video and the passing criteria. We recommend that you practice repeatedly, following these instructional videos until you are confident that you have mastered the knowledge and skills required for each goal. In general, it takes approximately 30 min to complete the practice.

| **Goal 1: High-quality CPR**  You should be able to perform 1 minute of uninterrupted compressions-only CPR with a QCPR score of at least 80% in the compression depth, release, and rate (without viewing real-time QCPR feedback). | **Goal 2: AED operation**  You should be able to operate the AED properly and avoid bystanders touching the manikin during defibrillation. | **Goal 3: Adult BLS sequence**  You should be able to complete the adult BLS sequence and perform 2 minutes of high-quality CPR with a QCPR score of at least 80% in the compression depth, release and rate (without viewing real-time QCPR feedback). |
| --- | --- | --- |


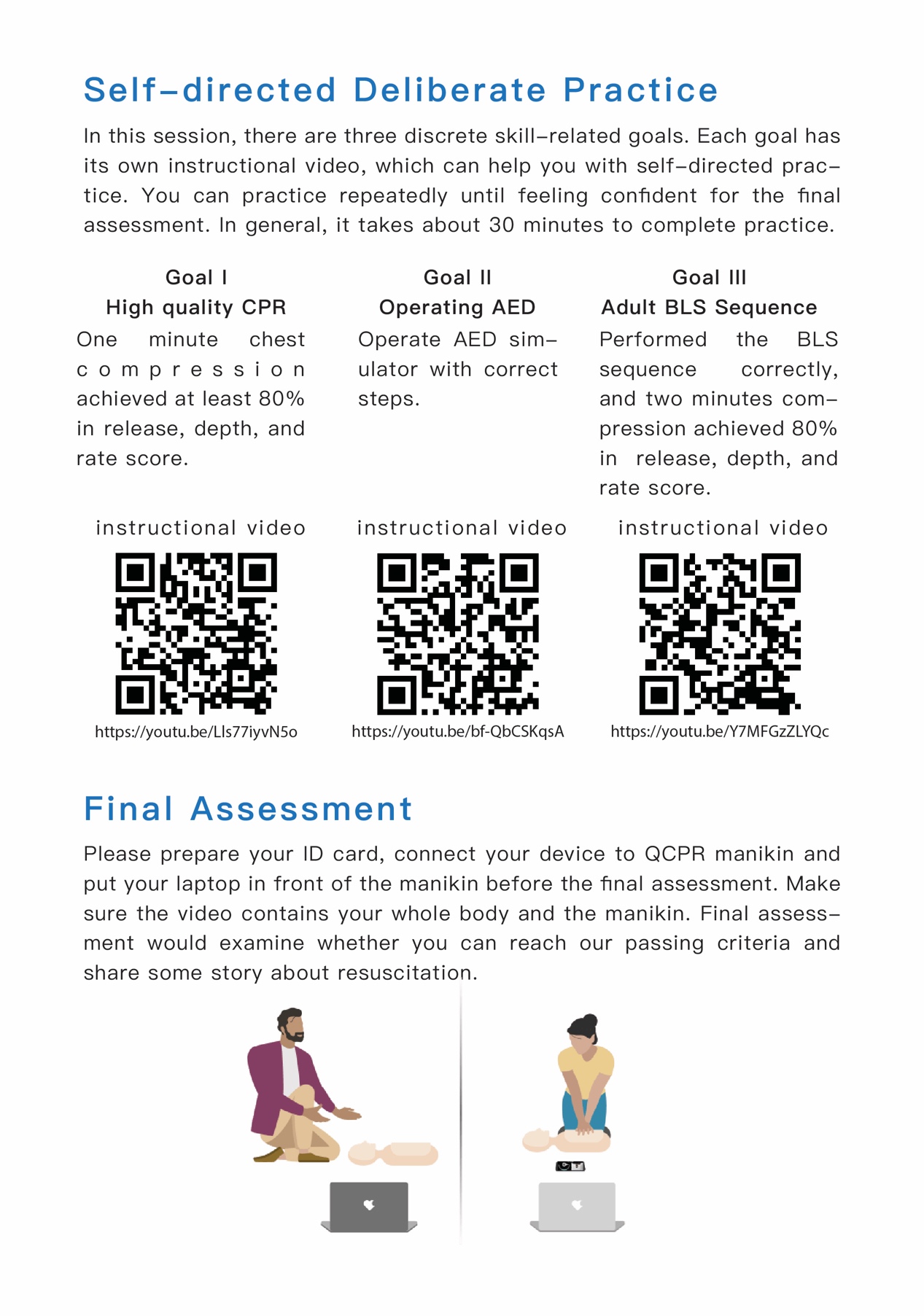


**Online Final Assessment**

We will evaluate your performance in this section, using Google Meet. Before that, please have your ID card ready, and ensure that the manikin and QCPR training app are ready to use. The videoconferencing camera should clearly capture movements while performing BLS and CPR on the manikin. After the test, the results of the QCPR score will be presented. The course instructor will provide feedback on your performance.
