# Supplementary material for: The Effectiveness of Online-Only Blended Cardiopulmonary Resuscitation Training: Static-Group Comparison Study
Source: J Med Internet Res. 2023 Apr 5;25:e42325. doi: 10.2196/42325 (PMC10131976; doi:10.2196/42325)
Supplement: Multimedia Appendix 3 [file jmir_v25i1e42325_app3.docx]

**Appendix 3: Questionnaire after the BLS course**

**Informed Consent**

1. Do you agree to your test results and questionnaire responses (excluding your name) being used for academic research or publication?
   1. Disagree
   2. Agree

**Participants’ background**

1. What is your main motivation for taking this BLS course?
   1. Personal factors (e.g., curiosity, self-improvement, public welfare)
   2. Factors related to relatives and friends (e.g., someone around you has a heart disease)
   3. Educational factors (e.g., CPR certification is needed for school graduation)
   4. Occupational factors (e.g., you are working as a healthcare provider, lifeguard)
   5. Personal interest (e.g., you are planning to take a professional license that needs CPR skills)
2. How long has it been since you last attended a similar CPR training (including hands-on practice), before taking this BLS course?
   1. There were no previous training courses.
   2. Less than 6 months
   3. 6 to 12 months
   4. 12 to 24 months
   5. More than 24 months
3. Have you ever performed CPR or AED on a real person?
   1. Yes
   2. No

**Self-evaluation of Deliberate Practice**

1. How much time did you spend in deliberate practice before taking the final online course assessment?
2. Less than 30 minutes
3. 30 to 60 minutes
4. 60 to 90 minutes
5. 90 to 120 minutes
6. More than 120 minutes
7. At the beginning of the deliberate practice, how would you rate your performance? Did you meet the three learning goals?
   1. Far below average
   2. Below average
   3. Average (i.e., met passing criteria)
   4. Above average
   5. Far above average
8. Before completing the final online assessment, how would you rate your performance? Did you meet the three learning goals?
   1. Far below average
   2. Below average
   3. Average (i.e., met passing criteria)
   4. Above average
   5. Far above average

**Self-evaluation of the BLS course**

1. After the BLS course, do you think it is more diﬃcult than expected?
   1. Totally disagree
   2. Partly disagree
   3. No comment
   4. Partly agree
   5. Totally agree
2. After the BLS course, has your knowledge of CPR+AED increased?
   1. Totally disagree
   2. Partly disagree
   3. No comment
   4. Partly agree
   5. Totally agree
3. After the BLS course, has your conﬁdence on CPR+AED increased?
   1. Totally disagree
   2. Partly disagree
   3. No comment
   4. Partly agree
   5. Totally agree
4. After the BLS course, has your willingness to perform CPR on a stranger
    increased?
   1. Totally disagree
   2. Partly disagree
   3. No comment
   4. Partly agree
   5. Totally agree
5. After the BLS course, has your willingness to perform mouth-to-mouth
    ventilation on a stranger increased?
   1. Totally disagree
   2. Partly disagree
   3. No comment
   4. Partly agree
   5. Totally agree
6. After the BLS course, has your willingness to operate the AED on a stranger
    increased?
   1. Totally disagree
   2. Partly disagree
   3. No comment
   4. Partly agree
   5. Totally agree
7. After the BLS course, will you refuse to perform BLS on strangers?
   1. Totally disagree
   2. Partly disagree
   3. No comment
   4. Partly agree
   5. Totally agree
8. After the BLS course, did you realize that the LittleAnne manikin was effective in CPR + AED training?
   1. Totally disagree
   2. Partly disagree
   3. No comment
   4. Partly agree
   5. Totally agree
9. After the BLS course, was self-directed learning found to be effective
    in CPR+AED training?
   1. Totally disagree
   2. Partly disagree
   3. No comment
   4. Partly agree
   5. Totally agree
10. After the BLS course, did you realize that online assessment was effective
     in CPR + AED training?
    1. Totally disagree
    2. Partly disagree
    3. No comment
    4. Partly agree
    5. Totally agree

**General information**

Name:

Sex:
 Age:
 Occupation:
